# The influence of facility and home pen design on the welfare of the laboratory-housed dog

**DOI:** 10.1016/j.vascn.2016.09.005

**Published:** 2017

**Authors:** Laura E.M. Scullion Hall, Sally Robinson, John Finch, Hannah M. Buchanan-Smith

**Affiliations:** a Behaviour and Evolution Research Group, Psychology, Faculty of Natural Sciences, University of Stirling, Stirling FK9 4LA, UK; b Drug Safety Metabolism, Innovative Medicines and Early Development Biotech Unit, Alderley Park, Macclesfield, Cheshire SK10 4TG, UK; c Charles River Laboratories, Elphinestone Research Centre, Tranent, UK

**Keywords:** Welfare, Laboratory-housed dog, 3Rs, Home pen design, Husbandry, Facility design, Housing, Enrichment, Strain

## Abstract

We have an ethical and scientific obligation to Refine all aspects of the life of the laboratory-housed dog. Across industry there are many differences amongst facilities, home pen design and husbandry, as well as differences in features of the dogs such as strain, sex and scientific protocols. Understanding how these influence welfare, and hence scientific output is therefore critical. A significant proportion of dogs' lives are spent in the home pen and as such, the design can have a considerable impact on welfare. Although best practice guidelines exist, there is a paucity of empirical evidence to support the recommended Refinements and uptake varies across industry. In this study, we examine the effect of modern and traditional home pen design, overall facility design, husbandry, history of regulated procedures, strain and sex on welfare-indicating behaviours and mechanical pressure threshold. Six groups of dogs from two facilities (total n=46) were observed in the home pen and tested for mechanical pressure threshold. Dogs which were housed in a purpose-built modern facility or in a modern design home pen showed the fewest behavioural indicators of negative welfare (such as alert or pacing behaviours) and more indicators of positive welfare (such as resting) compared to those in a traditional home pen design or traditional facility. Welfare indicating behaviours did not vary consistently with strain, but male dogs showed more negative welfare indicating behaviours and had greater variation in these behaviours than females. Our findings showed more positive welfare indicating behaviours in dogs with higher mechanical pressure thresholds. We conclude that factors relating to the design of home pens and implementation of Refinements at the facility level have a significant positive impact on the welfare of laboratory-housed dogs, with a potential concomitant impact on scientific endpoints.

## 1 Introduction

There are two crucial reasons to ensure the most humane use of dogs in scientific research: our ethical obligation to prevent suffering, and our scientific need to ensure that they are valid, reliable and predictive models. Legislative (e.g. European Directive 2010/63/EU) and ethical (e.g. Russell & Burch, 1959, the 3Rs) guidelines provide frameworks within which animals can be used in scientific research, however there remains a paucity of quantitative data on which to base best practice in the dog. The Refinement ‘R’ of the 3Rs (Replacement, Reduction, Refinement) refers to the minimising of harms and promotion of positive states across the lifecycle of the animal (Buchanan-Smith et al., 2005). The positive impact of Refinements to housing, husbandry practices and regulated procedures on data output has been demonstrated in several laboratory housed species such as rodents (Everds et al., 2013); primates (Tasker, 2012); and dogs (Hall, 2014), however Refinement uptake varies across industry.

Global dog use remains high (∼100,000 per year, Hall, 2014), yet the implementation of known Refinements varies considerably across industry and between countries. As the predominant use of dogs is the development of new medicines, it is critical to increase our understanding of effective Refinements.

### 1.1 Home pen design

**Fig. 1:**
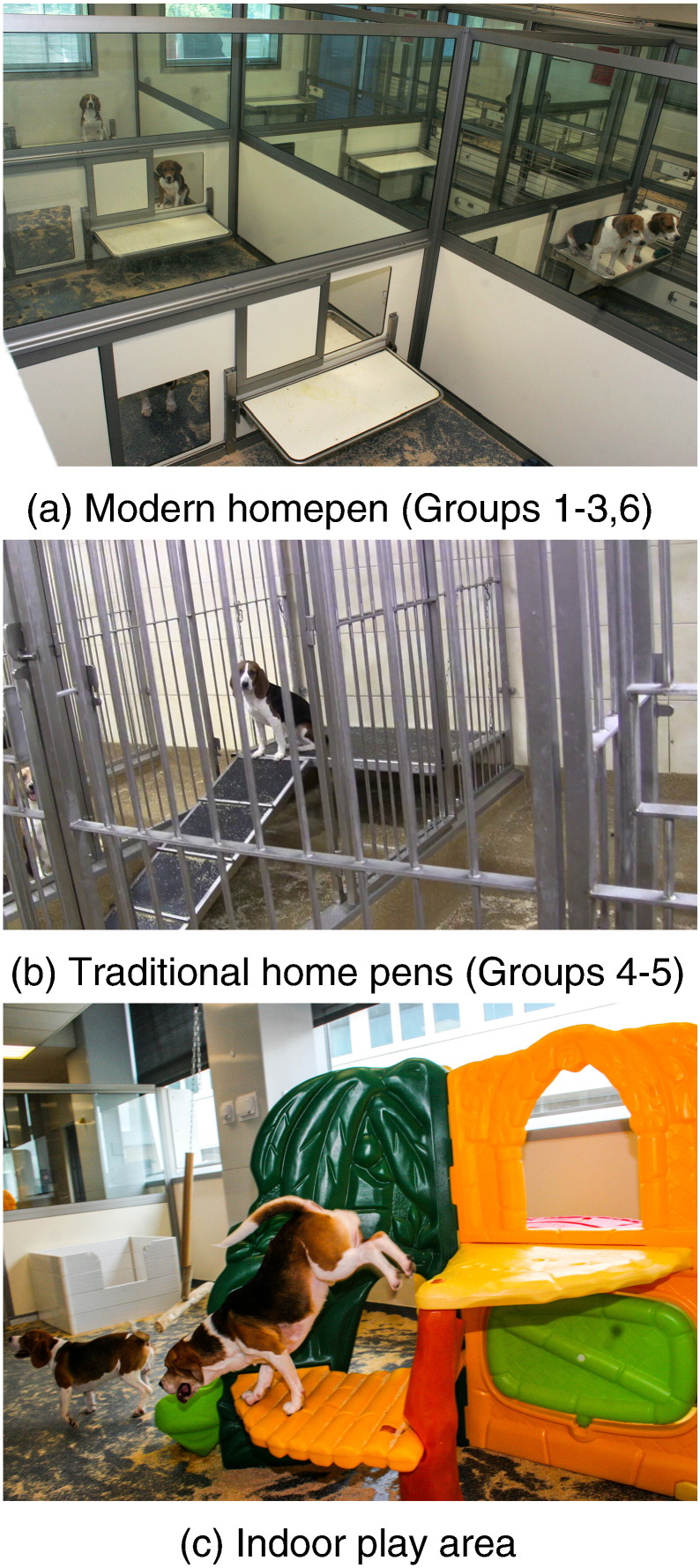
The design of dog (a) modern home pens (Groups 1–3 and 6), (b) traditional home pens and (c) indoor play areas. Aspects of good practice in the modern home pens can be seen, such as increased visibility for dogs and staff, horizontal bars to prevent “paddling”, a choice of locations, exit points and ledges within the pen, and the provision of climbing frames and toys in the play areas.

The design of the home pen and animal room (the area which includes home pens, corridors and any indoor play areas) may be one of the most crucial Refinements for dog welfare, however it has received little scientific attention since the 1990s, since when legislative minimum standards have improved. Dogs will spend the majority of the day in the home pen, so its design will have a considerable impact on welfare. EU legislation mandates a minimum pen size of 2.25 m^2^ per dog (10–20 kg) when group housed and 4 m^2^ when singly-housed, while other legislation (e.g. National Research Council, 2011, in the USA) mandates much smaller minimums, e.g. 0.74 m^2^ for dogs of a similar size. Despite industry moving towards modern dog unit and home pen design (see Fig. 1a) much of the supporting evidence for the benefits of their implementation remains anecdotal. Factors which are considered important for home pen design include visibility for dogs and staff, choice of resting places or platforms, size, ease of entry for staff, ease of partitioning dogs, and use of noise reducing materials (Hubrecht et al., 1992, Prescott et al., 2004, Sales et al., 1997). Lack of visibility or noise-reducing materials can cause allelomimetic barking which can lead to considerable noise which is detrimental to both dogs and staff (Prescott et al., 2004, Sales et al., 1997). Further illustrations of a modern home pen design can be found in Hall, Buchanan-Smith, Robinson, and Prescott (2015a).

### 1.2 Environmental enrichment

Environmental enrichment (EE, the provision of items or opportunities which enhance the well-being of captive animals and promote desirable behaviours (Buchanan-Smith, 2010)) is commonly provisioned as a Refinement to laboratory-housed dogs, and is recommended in both legislation and good practice guidelines. However, in order to act as Refinements, the enrichment items must improve the welfare of the dogs. Appropriate enrichment provides opportunities for animals to make choices, increasing their ability to maintain homoeostasis or to control social interactions (R. C.Hubrecht, 2014). Given the time spent in the home pen, providing suitable EE should be considered a critical Refinement for the laboratory-housed dog.

Novel toys, particularly those which can be chewed, are of interest to dogs (Hubrecht, 1993, Hubrecht, 1995) and can result in positive changes in behaviour (Hall, 2014). Separate indoor and outdoor play areas are also recommended (see Fig. 1c). The facilities studied in this paper differed in terms of the EE available to dogs (see Table 1).

### 1.3 Training for dogs and positive staff contact

Training dogs with positive reinforcement is a necessary component of smoothly-run animal units. Contact with staff is an unavoidable aspect of the environment, with staff responsible for pleasant events such as access to play areas, toys and feeding and also for carrying out regulated procedures or other unpleasant events (Balcombe, Barnard, & Sandusky, 2004). As a result, encouraging positive staff contact can discourage negative associations with staff members (Laule, 2010, Prescott et al., 2004). The facilities studied in this paper differed in terms of the provision of training and staff contact provided to dogs, see Table 1 for details.

### 1.4 Measuring welfare in the dog

‘Welfare’ has many uses in common language, but must have an objective definition in scientific use not influenced by moral or ethical considerations (Broom & Kirkden, 2004), and which concentrates on empirical evidence. Welfare can be understood in terms of physical health, and in terms of subjective experience. Broom (1986) describes welfare as a term which describes an individual's state in relation to its attempts to cope with a situation; therefore welfare does not reflect external circumstances but rather how effectively an individual is coping with them and the resulting impact on (evolutionary) fitness. It is well accepted that ‘welfare’ is a continuum from negative to positive, rather than a desirable condition. Behaviour can be thought as the ‘gold standard’ of welfare assessment, as it can be measured instantly, non-invasively, without the need for specialist equipment. However, in isolation, behaviour provides little information about the internal state of animals. The behavioural measures employed in this study are derived from a welfare assessment framework created for the laboratory-housed dogs (Hall, 2014, Hall et al., 2015) and which includes behaviour, affective state, cardiovascular output and mechanical pressure threshold.

Age, sex, strain and exposure to licensed procedures vary between the dogs studied in this paper. Although there is no consistent evidence of a welfare impact of age or sex in dogs, in general it is argued that younger animals which have experienced fewer adverse events have more positive welfare (Parker & Maestripieri, 2011), while some male animals may be more susceptible to stress (e.g. Dalla et al., 2005, Kaplan et al., 2009, rats and dominant male macaques respectively). Cumulative suffering (Honess & Wolfensohn, 2010) has been proposed as a model to describe the negative effects of repeated regulated procedures, however evidence is lacking in the dog. Therefore we include factors of strain, sex and history of regulated procedures in our analysis of welfare. It was not possible to analyse age as a separate factor in this study due to confounding factors of home pen design and history of regulated procedures between dogs of different ages.

The affective state of nonhuman animals can be assessed by judgement bias testing (Mendl, Paul, & Chittka, 2011), a protocol which has shown consistent results in many species, including the laboratory-housed dog (Hall, 2014). Animals with positive judgement biases have been shown to have greater tolerance for pain or mechanical pressure (e.g. Hall, 2014, Villemure and Bushnell, 2002) In this study we also recorded sensitivity to physical pressure using a mechanical algometer (TopCat Metrology Prod). This test of sensitivity to physical pressure (mechanical pressure threshold, MPT) is similar to pain threshold. Nociception (pain sensitivity) is known to vary with affective state in a variety of human and nonhuman species (e.g. Klauenberg et al., 2008, Villemure and Bushnell, 2002). We previously found that dogs exhibiting positive judgement biases had higher MPTs ( >12.5 N with an 8 mm tip), exhibited more restful behaviour in the home pen and more stable heart rate and blood pressure. In this study, we examine behaviour, mechanical pressure threshold and sound levels in relation to housing in contrasting styles of home pens. We highlight the importance of monitoring welfare on an ongoing basis, as some negative welfare indicators (e.g. alert behaviour, interacting with the environment, high posture) may be seen in the normal behavioural repertoire in response to disturbance in the environment, however their ongoing display in the absence of stimuli reflects negative welfare, which is reflected in our Welfare Assessment Framework (Hall, 2014).

### 1.5 Aims

We aimed to determine the impact of a number of factors on the welfare of laboratory-housed dogs by comparing two facilities in which home pen design, environmental enrichment, staff contact and training differed. We hypothesised that behaviour will indicate more positive welfare in (1) dogs housed in the purpose-built modern facility compared to the traditional facility, (2) in the modern home pen design compared to the traditional home pen design and (3) in those with no history of regulated procedures. We did not predict differences by sex and strain. We also hypothesised that (4) dogs with a mechanical pressure threshold over 12.5 N will show more behavioural indicators of positive welfare.

## 2 Materials and methods

### 2.1 Subjects and facilities

There were six groups in total (n = 46), three groups in each of Facilities A and B. Dogs were selected from a convenience sample of availability at the time of data collection. No dog was undergoing regulated procedures during our data collection. Several of the potential Refinements outlined in the introduction differed between Facilities A and B; Table 1 provides descriptions of the housing and husbandry for each group within Facilities A and B, and demographic details of the dogs within each group. Note that although Groups 1 and 2 had histories of long-term use in regulated procedures, Group 1 had been subject to more intensive use and procedures up to and including ‘moderate’ severity, while Group 2 experienced infrequent, short use and procedures which did not exceed ‘mild’ severity (see European Directive 20-10/63/EU for categorisation of severity bands). All dogs were housed in pairs or trios in interlinked home pens, with one dog per home pen. Each animal room contained between 10 and 32 home pens with a corridor separating pens, with male and female dogs housed on opposite sides of the room; in addition some animal rooms had an indoor play area (Groups 1–3 & 6 only). All animal rooms were on a 12 h light-dark cycle and dogs were fed once daily with a 300–350 g ration of SDS Diet dog food.

### 2.2 Behavioural observations

Behaviour was scored using a mixture of instantaneous sampling with a 30-second interval for behavioural states, and continuous sampling for behavioural events (see Martin & Bateson, 2007, for description of sampling methods). The coding scheme is listed in the supplementary materials; this includes behaviours which were scored but not reported due to low incidence (less than 5% of time, or less than 5 per hour). Behaviours are described as positive or negative welfare indicators, based upon the context (e.g. interacting with the environment is classed as negative as it is related to agitation) and frequency of appearance (e.g. some normal behaviour are classified as negative due to high occurrence) in which we are using them (see Hall, 2014).

All observations were recorded remotely using digital camcorders, with the exception of Group 1, whose observations were recorded via ceiling-mounted video cameras. The positioning of the CCTV camera's in Group 1’s home pens obscured some behavioural events relating to the face such as lip smacking, which may have affected the recorded rate of these behaviours. Other behaviours were unaffected. Recordings were made between approximately 7.30 h and 11 h, depending on facility. Ten five-minute observations were selected at least 20 min after recording began to allow dogs to settle after the experimenter left the animal room. It was not possible to blind the observer to group.

### 2.3 Mechanical pressure threshold

Testing was conducted using the TopCat Metrology ‘Prod’, a mechanical algometer which applies pressure at a rate of 2 N s^ −1^ and records mechanical pressure threshold in Newtons (N). The dogs were unrestrained and able to move away from the pressure, at which point the MPT reading was recorded by the device. Following the protocol used in Hall (2014), dogs were individually removed to a quiet room where three readings were taken from the centre of the back. This was repeated on a further two days, giving a total of nine readings in Newtons (N) for each dog. MPT data collection was evaluated by a Home Office Inspector and determined not to constitute a regulated procedure.

### 2.4 Sound levels

Sound levels were recorded in the animal rooms of Groups 4, 5 and 6 (Facility B) using a digital hand held sound meter. The experimenter walked to the centre of the room and recorded the sound level, in decibels, 30 s after entering the room. Readings were taken on three days and the mean value is presented.

### 2.5 Data analysis

Behavioural coding was conducted using The Observer XT 10.5, using instantaneous (behavioural states) and all-occurrence (behavioural events) sampling, and the duration of behavioural states and the rate per minute of behavioural events over the observed time were calculated.

Data were exported to a spreadsheet as proportions (states) or rate per minute (events). Many of the proportional behavioural data were found not to be normally distributed. An angular transformation was performed using the formula

(1) degrees(asin(√x))

where *x* is the original proportion. This transformation brought much of the data into normal distribution and allowed the use of parametric tests. This transformation also resulted in data being presented as estimated percentages of total time, to allow for ease of interpretation. The rate of behavioural events was also transformed to give a rate per hour (*x* * 60, where *x* is the original rate per minute) to allow data to be more clearly presented. Normally-distributed data were analysed using mixed design ANOVAs, with observation as a within-subjects factor, and Facility, group, sex or strain as between-subjects factors. Non-parametric data were analysed using Mann-Whitney *U* tests for between-subjects comparisons and Kruskal-Wallis tests for within-subjects tests. MPT data were analysed using a mixed design ANOVA, with measurement (9 levels) as a within-subjects factor, and Facility and group as between subject factors. The level of significance applied was *α* < 0.005.

### 2.6 Ethics

No regulated procedures, as defined in A(SP)A (Home Office, 1986, updated 2012) were conducted in this study. All dogs were housed in accordance with the relevant codes of practice. Ethics approval was granted by the Psychology, University of Stirling Ethics Panel before the study began, and study conduct was overseen by care staff. The use of the MPT device was given permission by a Home Office Inspector, with use up to a maximum of 28 N classed as a non-regulated procedure.

## 3 Results

### 3.1 Between-facility comparison

**Fig. 2:**
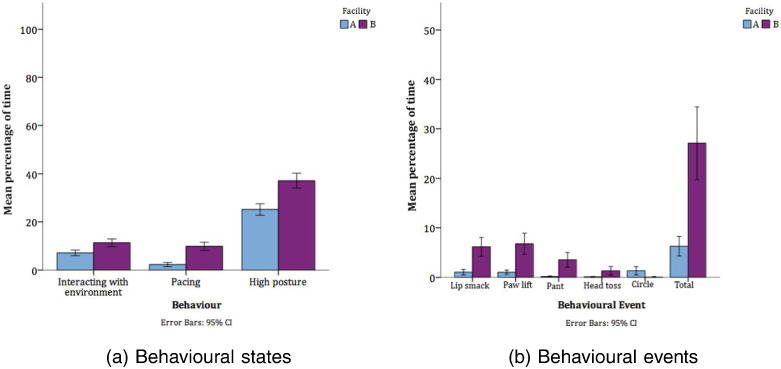
Significant differences in behavioural states and events between Facilities A and B (n=46).

Facilities A and B were compared to assess the overall impact of facility on welfare. Significant differences in behaviour between dog in these facilities are presented in Table 2, Fig. 2. All differences in behaviour were in negative welfare indicating behaviours and indicate more positive welfare in Facility A, the purpose-built modern facility.

### 3.2 Within-facility comparison

#### 3.2.1 Facility A

**Fig. 3:**
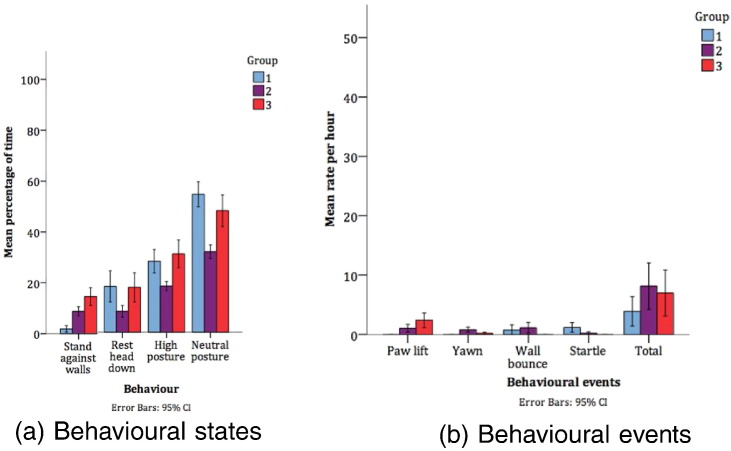
Significant differences in behavioural states and events between Facility A Groups 1–3 (n=22).

The comparisons within Facility A allowed us to investigate the overall effects of husbandry and history of regulated procedures on welfare, within a purpose-built modern facility. Groups 1–3 were housed in home pens of similar design, although differed in terms of staff contact and past use in regulated procedures. Groups 1 and 2 had a history of long-term use in regulated procedures, while Group 3 were naive to regulated procedures. Significant differences in behaviour of comparisons between Groups 1–3 are presented in Table 3, Fig. 3. These results show differences in positive and negative welfare indicating behaviours, with Group 2 (long-term but infrequent use) exhibiting more positive welfare (e.g. more resting behaviour, less high posture) but also more stereotypic behaviours than Groups 1 and 3.

#### 3.2.2 Facility B

**Fig. 4:**
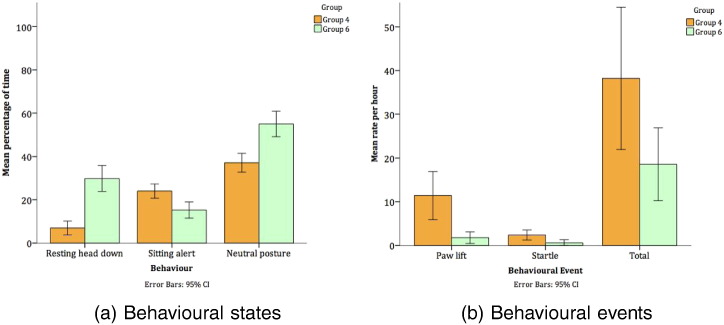
Significant differences in behavioural states and events between Groups 4 (traditional home pen design, n=8) and 6 (modern home pen design, n=8).

Comparing groups within Facility B allowed us to examine the effect of a modern home pen design (Group 6) against traditional home pen design (Group 4) where other factors were the same within a traditional facility. Significant differences in behaviour are shown in Table 4 and Fig. 4. Each of these behavioural differences suggests more positive welfare in Group 6 (modern home pen design).

##### Noise levels

Average sound level was calculated in each animal room in Facility B from three measurements taken on separate days. Noise was lowest on average in Group 6 (modern home pen design, Strain II) at 77.6 dB and highest in Group 5 (traditional home pen design, Strain III) at 105.7 db. Group 4 (traditional home pen design, Strain II) was intermediate at 99.7 dB.

### 3.3 The effects of sex and strain

The behaviour of male and female dogs from Groups 2–6 was compared to determine the effects of sex on welfare (n = 38), while Groups 4 and 5 within Facility B were compared to determine if behaviour varied between Strains II and III where other factors were identical (n = 16).

**Fig. 5:**
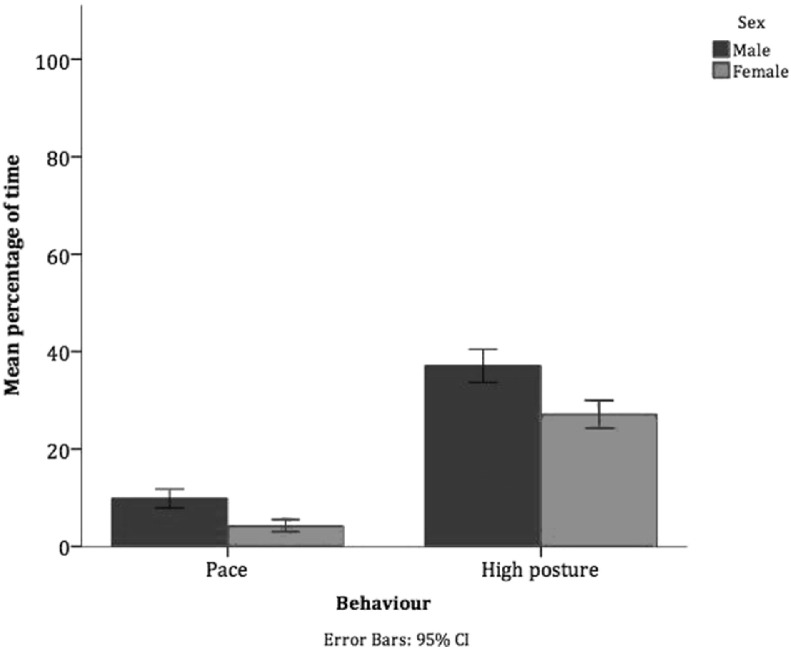
Significant differences in behavioural states between the sexes (n=38).

When comparing the sexes, Group 1 were excluded as the group consisted of only male dogs and had been subjected to more regulated procedures than other groups. Male dogs (n = 19) exhibited significantly more pacing, high posture and total stereotypies when compared to female dogs (n = 19), indicating that male dogs had more negative welfare (Table 5, Fig. 5). Several significant interactions were found between Group and sex which are also shown in Table 5; these interactions show either more negative welfare in male dogs compared to female dogs within a group, or more variable welfare between males in different groups. No pattern of welfare indicating behaviours was found when comparing behaviour between Groups 4 and 5.

### 3.4 Mechanical pressure threshold

Mechanical pressure threshold (MPT) was compared between dogs from Facilities A and B using a mixed design ANOVA, and no significant difference was found (P  > 0.05). There was also no effect of group on MPT (P  > 0.05).

**Fig. 6:**
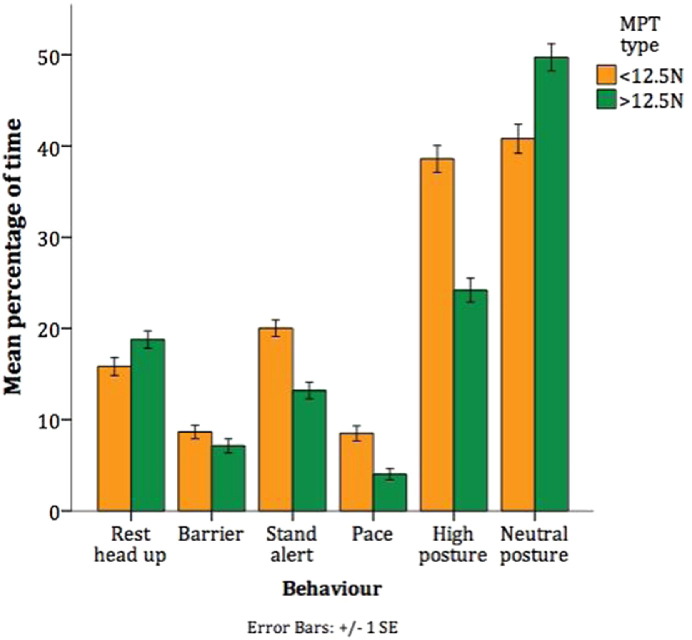
Significant differences in behaviour between dogs by mechanical pressure threshold (n=44).

We have previously found differences in welfare indicating behaviours between dogs with MPT above or below 12.5 N. As such, behaviour for all dogs (n = 44) was compared between those with MPT below 12.5 N (n = 25) and above 12.5 N (n = 21) using a repeated-measures ANOVA. Significant differences in several key behaviours were found between high and low MPT dogs (Table 6, Fig. 6), suggesting more positive welfare in dogs with MPT above 12.5 N.

## 4 Discussion

Our findings broadly support our hypotheses. We hypothesised that behavioural indicators of welfare would indicate more positive welfare in dogs (1) housed in a purpose-built modern facility, (2) in modern home pen design, (3) dogs naive to regulated procedures and (4) in those with higher MPTs. We did not find any consistent differences in welfare between dogs with different histories of regulated procedures within Facility A. We also found little effect of strain or age on welfare, however we did find several indicators of more negative welfare or more variable welfare in male dogs. These findings are further discussed below.

### 4.1 Between-facility comparisons

There were a total of nine significant differences in behaviour between Facilities A and B; in each of these differences, dogs in Facility A were found to be exhibiting more positive welfare. Negative welfare indicators such as pacing and stereotypies were considerably higher in Facility B. Measures of vigilance and disturbance in the environment such as high posture and interacting with the environment were also higher in Facility B. As noted earlier, interacting with the environment is interpreted as negative because it relates to vigilance and agitation. The provision of modifications such as modern home pen design, EE and indoor and outdoor play areas, and regular training (as in Facility A) are frequently recommended in guidelines (e.g. Prescott et al., 2004). The benefits of a purpose-built facility with modern home pen design are evident from our data. A number of factors varied between dogs in Facilities A and B which make it difficult to identify the individual effects of Refinements, it is likely to be their combination that positively impacts on welfare.

### 4.2 The effects of husbandry and history of regulated procedures (Facility A)

We predicted that welfare would be most positive in younger dogs with no history of regulated procedures (Group 3). There is no clear pattern of behavioural differences between Groups 1–3 which relate to dogs' history of regulated procedures (greatest in Group 1 and absent in Group 3). Group 2 spent more time resting and less time with high posture than the other groups, but also spent less time in a neutral posture, suggesting that overall Groups 1 and 3 were less relaxed than Group 2. Group 2 had a small, constant group of care staff who were regularly present. A variety of staff passed through Group 3’s animal room and there was a large team care staff, while Group 1 had only 1 member of care staff and little activity in the animal room other than regulated procedures. An absence of care staff except for feeding and regulated procedures (Group 1) or sporadic appearances (Group 3) is likely to have contributed to increased vigilance because of the unpredictable nature of staff appearances (Bassett & Buchanan-Smith, 2007). This is reflected in the higher rate of startling in Group 1.

### 4.3 The effects of home pen design (Facility B)

When comparing Groups 4 and 6, which consisted of identical groups of dogs housed in contrasting home pen designs in Facility B, several significant differences were found. The pattern was consistent, with Group 6 (modern) displaying more positive welfare indicating behaviours and fewer negative welfare indicating behaviours then Group 4 (traditional). For example, time resting more than doubled while the rate of stereotypic behaviours was more than halved. Other behavioural indicators of negative welfare such as pacing and standing alert were not lower in Facility B's modern home pen design. However it is clear that this style of home pen and animal room design promotes better welfare than the traditional design, while facility level Refinements (such as in Facility A) may be more effective still, as seen when comparing Facilities A and B. Noise levels measured in animal rooms were lower in Group 6’s than Group 4’s animal room on each day. Modern home pen designs are often advocated on the basis of reducing allelomimetic barking and overall noise caused by barking (as recommended in Prescott et al., 2004). Given the negative impact of noise exceeding 85–90 dB not only on dog welfare (Coppola et al., 2006, Sales et al., 1997), but on staff health (Turner, Parrish, Hughes, Toth, & Caspary, 2005), our findings of reduced barking noise between two identical groups of dogs housed in contrasting home pen designs support the provision of modern home pen design to reduce barking noise.

### 4.4 Sex and strain

There is a lack of clear evidence in the literature of an effect of sex or age on dog welfare and so we had no clear predictions. As behaviour and physiology differ between strains in other laboratory-housed species we also compared welfare between commonly-used strains.

When comparing two groups of Facility B dogs (Groups 4 and 5) housed in identical conditions which differed only in strain, we found few differences in behaviour and no effect on the more clear indicators of welfare such as alert and resting behaviour. We also found no effect of sex or strain on MPT but did find some effect on behaviour. When we compared the sexes, many of the behavioural differences were attributable to more negative welfare in male dogs or inconsistent behaviour between the groups in male dogs. This suggests that male dogs may be less able to maintain homoeostasis and may be at greater risk of suffering negative welfare. The natural history of dogs (e.g. Spotte, 2012) suggests that young male dogs would not normally live in constant groups and this artificial arrangement may cause stress for some individuals. As most time is spent in home pens, it is important to monitor welfare here, however as we did not observe dogs during regulated procedures or other aversive events, this effect of sex may be more evident and as such, welfare should be monitored during and after these events. Home pen and facility design appear to explain the greatest difference in welfare of the factors investigated in this paper, therefore these should be considered more important to welfare than sex, strain or age. There were also a number of factors which differed between the six Groups, which may also have influenced behaviour in addition to sex and an investigation of matched groups which differ only in sex may be beneficial to elucidate the influence of sex on welfare. However as male dogs have exhibited more variability in behaviour in this study, they may be more vulnerable to stress and should be monitored closely. The greater behavioural variation at baseline levels may also lead to greater residual variation in scientific endpoints, requiring larger sample sizes to achieve significance (i.e. opposing the Reduction R of the 3Rs).

### 4.5 MPT and behavioural differences

We hypothesised that dogs with MPT  < 12.15 N would exhibit more behavioural indicators of negative welfare than dogs with MPT  > 12.15 N. We found greater positive welfare indicators (resting behaviour and neutral posture) and lower negative welfare indicators (alert behaviour and high posture) in dogs with MPT  > 12.15 N. There were no interactions with sex, strain, group or facility, suggesting that this effect is consistent across all dogs. It is surprising, given the differences in behaviour, that no difference in MPT was detected between the facilities.

Welfare varies in animals exposed to the same environment, based on past experience, genetics and rearing environment (Broom, 1991). This may explain why welfare varies within the groups in this study. The purpose of providing Refinements is to harmonise welfare by providing more opportunities for coping strategies. We previously found that these differences in welfare and mechanical pressure threshold were found in dogs which tested differently in cognitive bias testing, a measure of affective state in animals. We suggest that the individual differences in welfare may be influential (e.g. Koolhaas, 2008) in addition to the Refinements implemented. The provision of Refinements such as EE and positive staff contact may also explain why there are fewer differences between naive dogs and those having experienced long-term use in Facility A (Groups 1–3) than there are between naive dogs in Facility A (Group 3) and Facility B (Groups 4–6).

The provision of Refinements such as the implementation of a modern dog unit design or the provision of environmental enrichment harmonise welfare, rather than to standardise it by providing animals with choice and complexity. This harmonisation can result in decreased residual variation which is detrimental to scientific output (e.g. see Würbel, 2000).

### 4.6 Conclusions

We provide evidence that modifications commonly recommended in good practice guidelines for the laboratory-housed dog, particularly home pen design, environmental enrichment and inclusion of regular training and staff contact are important to promote positive welfare and Refine the lifetime experience of the dogs. The more positive welfare found in Facilities A compared to Facility B support the use of these Refinements at a facility level. The differences in welfare found between traditional and modern home pen designs in Facility B also support the value of a modern home pen and animal room design (i.e. the inclusion of an indoor play area adjacent to home pens). Few differences were found between strains, suggesting that welfare indicating behaviours do not vary significantly between these strains, however we did find evidence of more negative and more variable welfare in male dogs. Differences were found in welfare indicators between dogs with higher or lower MPT (as determined by MPT testing), supporting the use of our Welfare Assessment Framework across strains and facilities. We recommend that the Refinements described here are implemented consistently across industry and suggest that factors such as home pen design should be included in the design of experimental studies.

## Figures and Tables

**Table 1:** Housing, husbandry and history details for both Facilities and all Groups.

Feature	Facility A			Facility B		
Group	1	2	3	4	5	6
Number (m/f)	8 (8/0)	8 (4/4)	6 (3/3)	8 (4/4)	8(4/4)	8 (4/4)
Age (mo)	20–32	25–42	18–25	6	6	8
Strain	I	I	I	II	III	II
Source: commercial supplier				✓	✓	✓
Bred on-site	✓	✓	✓			
Used in regulated procedures	✓	✓				
Home pen size	4.84 m^2^	4.84 m^2^	4.84 m^2^	2.5 m^2^	2.5 m^2^	2.5 m^2^
Minimum interlinked pen size per dog	9.68 m^2^	9.68 m^2^	9.68 m^2^	5 m^2^	5 m^2^	5 m^2^
Indoor play area	✓	✓	✓			✓
With climbing frames	✓	✓	✓			
Outdoor play area	✓	✓	✓			
Corridor exercise only				✓	✓	
Ledges per pair of pens	6	6	6	1	1	2
Extensive enrichment^a^	✓	✓	✓			
Care staff: single	✓					
Small team		✓		✓	✓	✓
Large changing team			✓			
Weekly table training	✓	✓	✓			
Weekly health checks	✓	✓	✓	✓	✓	✓

a Extensive enrichment comprised various chews and toys in home pen; all Facility B home pens had one suspended Kong and two Nylabones per pair of pens.

**Table 2:** Results of mixed-design ANOVA and Mann Whitney *U* tests, showing significant differences in behaviour between Facilities A and B.

Valence	Behaviour	*F*	df	P	Direction
Negative	Interacting with environment	12.931	1,44	0.001	B > A
	Pacing	17.958	1,44	<0.001	B > A
	High posture	5.274	1,44	0.027	B > A
	Total stereotypies	4.109	1,44	0.049	B > A
Valence	Behaviour	*U*		P	Direction
Negative	Lip smack	22,274.0		<0.001	B > A
	Paw lift	22,163.0		<0.001	B > A
	Pant	24,035.0		0.001	B > A
	Head toss	25,424.0		0.036	B > A
	Circle	24,828.0		<0.001	B > A

**Table 3:** Results of mixed-design ANOVA and Kruskal-Wallis tests, showing significant between-Group differences in home pen behaviour for Facility A (n=22).

Type	Behaviour	*F* (1,22)	P	Direction
Positive welfare indicator	Resting head down	4.450	0.013	Group 2 > Group 1 & Group 3
Negative welfare indicator	Standing against walls	18.431	<0.001	Group 3 > Group 1
Posture (−ve)	High posture	4.624	0.011	Group 1 & Group 3 > Group 2
(+ve)	Neutral posture	15.361	<0.001	Group 1 & Group 3 > Group 2
Type	Behaviour	*χ*^2^ (2)	P	Direction
Stereotypy (−ve)	Paw lift	26.095	<0.001	Group 3 > Group 2 > Group 1
	Yawn	16.979	<0.001	Group 2 > Group 3 > Group 1
	Wall bounce	27.791	0.006	Group 1 & Group 2 > Group 3
	Startle	6.954	0.31	Group 1 > Group 3
	Total	13.388	0.001	Group 2 & Group 3 > Group 1

**Table 4:** Results of mixed-design ANOVA and Mann Whitney *U* tests, showing significant differences in behaviour between Group 4 (traditional home pen design, n = 8) and Group 6 (modern home pen design, n = 8).

Type	Behaviour	*F*	df	P	Direction
Positive welfare indicator	Resting head down	12.001	1,22	0.004	Group 6 > Group 4
Negative welfare indicator	Sit alert	4.785	1,22	0.046	Group 4 > Group 6
Posture (+ve)	Neutral posture	9.141	1,22	0.009	Group 6 > Group 4
Stereotypy (−ve)	Total stereotypies	9.535	1,22	0.008	Group 4 > Group 6
Type	Behaviour	*U*		P	Direction
Stereotypy (−ve)	Paw lift	2367.5		<0.001	Group 4 > Group 6
	Startle	2726.0		0.003	Group 4 > Group 6

**Table 5:** Results of mixed-design ANOVA, showing significant differences and interactions in negative welfare indicating behaviours between and within the sexes (n=38), ♀=male, ♂=female.

Behaviour	*F*	df	P	Direction or interaction
Pacing	13.168	1,36	0.001	♀ > ♂
High posture	7.749	1,36	0.009	♀ > ♂
Total stereotypies	4.029	1,36	0.049	♀ > ♂
Interacting with environment	2.706	4,28	0.013	Group 2 ♀ > Group 2 ♂
Standing alert	5.129	4,28	0.012	Group 3 ♀ > Group 3 ♂
Pacing	2.195	4,28	0.001	Group 5 ♀ > Group 5 ♂
High posture	4.082	4,28	0.006	Group 5 ♀ > Group 5 ♂
Standing alert	5.129	4,28	0.006	Group 2 ♀ > Group 3 ♀
Pacing	2.195	4,28	0.049	Group 5 ♀ > Group 2 ♀
				Group 6 ♀ > Group 3 ♀
High posture	4.082	4,28	0.006	Group 5 ♀ > Group 2 ♀
Stereotypies	2.690	4,28	0.022	Group 4 ♀ > Group 6 ♀

**Table 6:** Results of mixed-design ANOVA, showing significant differences in behaviour between dogs with MPT above or below 12.5N (n=44).

Type	Behaviour	*F*	df	P	Direction
Positive welfare indicator	Resting head up	7.145	1,44	0.011	Higher when MPT > 12.15 N
Negative welfare indicator	Stand alert	4.676	1,44	0.036	Lower when MPT > 12.15 N
	Pacing	5.799	1,44	0.020	Lower when MPT > 12.15 N
Posture (−ve)	High posture	7.179	1,44	0.010	Lower when MPT > 12.15 N
Posture (+ve)	Neutral posture	5.572	1,44	0.023	Higher when MPT > 12.15 N
