# Supplementary material for: The influence of facility and home pen design on the welfare of the laboratory-housed dog
Source: J Pharmacol Toxicol Methods. 2017 Jan-Feb;83:21–9. doi: 10.1016/j.vascn.2016.09.005 (PMC5239769; doi:10.1016/j.vascn.2016.09.005)
Supplement: Supplementary file 1 — Supplementary material. [file mmc1.pdf]

Appendix

The coding scheme

Behaviours are listed and described, along with the sources of the description, in the supplementary information. Behaviours marked as †were scored but not analysed as they were exhibited for less than 5% of time (states) or fewer than five times per hour (events).

Table 7: Behavioural measures of positive welfare in the home pen

**Behaviour**	**Description**	**Source**
Resting head up	Sitting or lying, not apparently asleep but not orientated towards any stimulus	Beerda et al. (1998), Haverbeke et al. (2008)
Resting head down	Lying, may be apparently asleep, not orientated towards any stimulus	Beerda et al. (1998), Hubrecht et al. (1992), Spangenberg et al. (2006), Haverbeke et al. (2008)
Amicable	Lick, play, allogroom dog, often with tail wag	Hubrecht et al. (1992), Schipper et al. (2008), Horowitz (2002)
Play (social or self)	Bow, metaplay, may involve toys or other objects, bouncing gait, play face, wrestle, play chase	Hubrecht et al. (1992), Hubrecht (1993),Horowitz (2002) Spangenberg et al. (2006), Schipper et al. (2008),
†Calm locomotion	Walk, 4 Beat gait and 3 feet on the ground at any one time	Overall (2014)

Table 8: Behavioural measures of negative welfare in the home pen

Behaviour	Description	Source
†Autogrooming	Cleans self using mouth and/or paws	Beerda et al. (1998), Haverbeke et al. (2008)
†Digging	Using the paws to repetitively dig at a surface or object	Beerda et al. (1998)
†Destruction of environment	Using teeth or paws to tear or damage pen or objects	Beerda et al. (1998)
Interact with environment	Air sniffing, investigating pen or objects	Beerda et al. (1998)
Stand walls	Stands on hind legs with forelegs against wall	Beerda et al. (1998), Hubrecht et al. (1992), Hubrecht (1993), Spangenberg et al. (2006), Beerda et al. (1999), Haverbeke et al. (2008), Schipper et al. (2008)
†Aversive	Snarl,bite, defend, raise hackles	Hubrecht et al. (1992), Hubrecht (1993), Spangenberg et al. (2006), Horowitz (2002)
†Circling	Repetitive movement around pen	Beerda et al. (1998), Hubrecht et al. (1992), Hubrecht (1993), Beerda et al. (1999), Haverbeke et al. (2008),
Pace	Repetitive pacing, usually along a boundary	Hubrecht et al. (1992), Hubrecht (1993), Haverbeke et al. (2008)
Sit alert	Dog orientated towards stimulus while in a sitting position	Ley et al. (2008)
Stand alert	Dog orientated towards stimulus while in a standing position, usually accompanied by high posture	Ley et al. (2008)
Rapid locomotion	Trot, 2 Beat gait, diagonally opposite legs move together	Overall (2014)
†Crouch	Bent legs, body lowered towards ground	Beerda et al. (1998)
†Tremble	Clear shivering of the body	Beerda et al. (1998)
†Vocalise	Barking, growling, soft whining, loud whining, low pitched or high pitched vocalisations, yelping.	Beerda et al. (1998), Haverbeke et al. (2008)

Table 9: Postural measures of welfare in the home pen

Posture	Description	Source
High (negative welfare indicator/context dependent)	Breed specific posture as shown under neutral conditions, with the addition of high tail, head and ear position	Beerda et al. (1998), Beerda et al. (1999)
Neutral (positive welfare indicator)	Breed specific posture as shown under neutral conditions	Beerda et al. (1998), Overall (2014)
Half-low (negative welfare indicator)	Two features from: low position of tail, backwards bending of ears, bent legs	Beerda et al. (1998), Beerda et al. (1999), Haverbeke et al. (2008)
Low (negative welfare indicator)	As above, all three features present	Beerda et al. (1998), Beerda et al. (1999), Haverbeke et al. (2008)
†Very low (negative welfare indicator)	As above, with body close to ground	Beerda et al. (1998), Haverbeke et al. (2008)
†Tail wag high (context dependent)	Repetitive movements with the tail held high	Beerda et al. (1998), Beerda et al. (1999), Haverbeke et al. (2008), Normando et al. (2009)
†Tail wag low (context dependent)	Repetitive movements with the tail held low	Beerda et al. (1998), Beerda et al. (1999), Haverbeke et al. (2008), Normando et al. (2009)

Table 10: Behavioural events indicating negative welfare in the home pen

**Behaviour**	**Description**	**Source**
Startle	Sudden jump in response to stimulus	Schipper et al. (2008)
Body shake	Whole body shivers, trembles	Beerda et al. (1998), Beerda et al. (1999), Schipper et al. (2008)
Oral behaviours	Includes tongue out, snout licking, swallowing, lip smacking	Beerda et al. (1998), Beerda et al. (1999), Haverbeke et al. (2008)
Paw lift	Sudden raising of one limb, usually foreleg, and usually in response to stimulus	Beerda et al. (1998), Beerda et al. (1999), Haverbeke et al. (2008), Schipper et al. (2008), Stephen & Ledger (2005)
Yawn	The mouth is opened wide and a long deep breath is taken	Beerda et al. (1998), Beerda et al. (1999), Haverbeke et al. (2008), Schipper et al. (2008)
Pant	Open mouth, rapid breathing with tongue extended	Beerda et al. (1998), Haverbeke et al. (2008)
Jump	All four limbs leave ground simultaneously	Hubrecht et al. (1992), Hubrecht (1993), Spangenberg et al. (2006)
Wall bounce	Dog jumps towards wall and contacts with limbs	Hubrecht et al. (1992), Hubrecht (1993)
Jerk	Sudden movement, usually away from a stimulus	Schipper et al. (2008)
Circle	Singular rapid movement around pen	Schipper et al. (2008)

References

Beerda, B., Schilder, M., Van Hooff, J., De Vries, H., & Mol, J. (1998). Behavioural, saliva cortisol and heart rate responses to different types of stimuli in dogs. *Applied Animal Behaviour Science*, *58*, 365–381.

Beerda, B., Schilder, M., Van Hooff, J., De Vries, H., & Mol, J. (1999). Chronic stress in dogs subjected to social and spatial restriction. I. Behavioral responses. *Physiology and Behavior*, *66*, 233–242.

European Union(2010). Directive 2010/63/EU of the European Parliament and of the Council of 22 September 2010 on the protection of animals used for scientific purposes. *Official Journal of the European Union*, *L 276/33*,

Haverbeke, A., Laporte, B., Depiereux, E., Giffroy, J.-M., & Diederich, C. (2008). Training methods of military dog handlers and their effects on the team's performances. *Applied Animal Behaviour Science*, *113*, 110–122.

Horowitz, A. C. (2002). *The behaviors of theories of mind, and a case study of dogs at play.* (Ph.D. thesis). San Diego: University of California

Ley, J., Bennett, P., & Coleman, G. (2008). Personality dimensions that emerge in companion canines. *Applied Animal Behaviour Science*, *110*, 305–317.

Normando, S., Corain, L., Salvadoretti,M., Meers, L., & Valsecchi, P. (2009). Effects of an enhanced human interaction program on shelter dogs' behaviour analysed using a novel nonparametric test. *Applied Animal Behaviour Science*, *116*, 211–219.

Overall, K. L. (2014). The ethogram project. Journal of Veterinary Behavior: Clinical Applications and Research, 9, 1–5.

Schipper, L. L., Vinke, C. M., Schilder, M. B., & Spruijt, B.M. (2008). The effect of feeding enrichment toys on the behaviour of kennelled dogs (*Canis familiaris*). *Applied Animal Behaviour Science*, *114*, 182–195.

Spangenberg, E. M. F., Bjorklund, L., & Dahlborn, K. (2006). Outdoor housing of laboratory dogs: Effects on activity, behaviour and physiology. *Applied Animal Behaviour Science*, *98*, 260–276.
